# Predilatation of a stenotic ostium of a bronchial artery, followed by embolization in recurrent hemoptysis

**DOI:** 10.4103/0971-3026.69363

**Published:** 2010-08

**Authors:** Mathew P Cherian, Pankaj Mehta, Tejas M Kalyanpur, Sandeep S Hedgire, Kaustubh Narsinghpura, K Venkatesh

**Affiliations:** Department of Radiology, Kovai Medical Center and Hospital, Avinashi Road, Coimbatore, Tamil Nadu – 641 014, India

**Keywords:** Bronchial artery embolisation, ostial stenosis, hemoptysis

## Abstract

Bronchial artery embolization may be the only life-saving procedure available in a patient presenting with massive hemoptysis. Rarely, selective catheterization of these vessels may be rendered difficult due to a stenotic ostium. This may result in closure of the vessel or absence of forward flow after the stenotic segment is crossed with a diagnostic catheter or a microcatheter. Further, it may also lead to recurrence of hemoptysis if the distal vessel and the prearteriolar bed are inadequately embolized. We describe a technique of selective cannulation of the stenotic vessel, dilatation of the stenosis and then successful embolization.

## Introduction

Bronchial artery embolization (BAE) has now established itself as the standard of care in life-threatening hemoptysis.[1] One cause of technical failure is ostial stenosis which may lead to any or all of the following problems. Firstly, the catheter tip may be unstable in position and may get dislodged during injection of the embolic agent, leading to inadvertent embolization of normal vessels.[2] Secondly, since effective embolization of distal vessels and the prearteriolar bed requires a good antegrade flow, a stenotic ostium may lead to absence of adequate forward flow and the embolic agent may get deposited in the proximal vessels. This may in turn result in collateralization of the distal bed by other vessels and consequent early recurrence of hemoptysis.[2] Thirdly, when the catheter crosses the tight stenosis, the hemodynamic effect may be very similar to that produced by wedging of the catheter. Injection of the embolic agent under pressure may open up collateral vessels which were not visualized and non-target vessels may be embolized. Maintaining adequate forward flow in the target vessels is of paramount importance to ensure successful embolization.[2]

## Materials and Methods

**Figure 1 (A,B):**
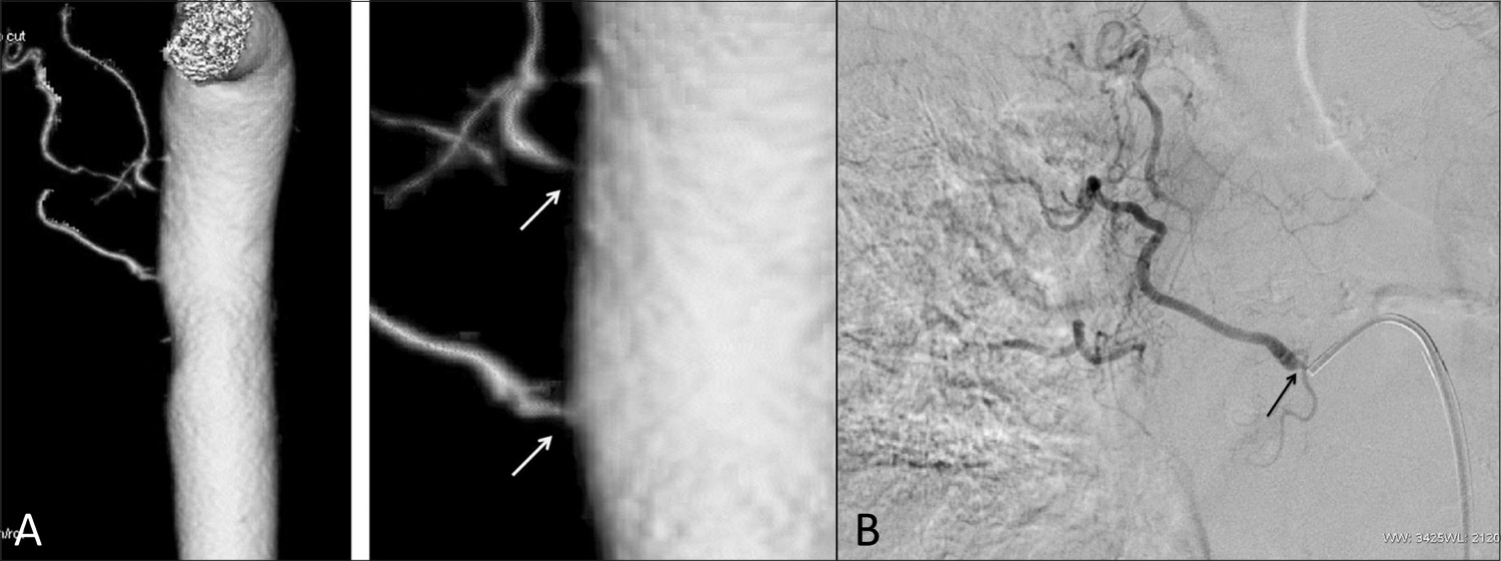
Bronchial artery angiograms. CT angiogram of the aorta (A) shows severe stenoses (arrows) at the ostia of the right bronchial arteries. DSA (B) shows severe ostial stenosis (arrow) in the culprit vessel that was not embolized at the first sitting

**Figure 2 (A-C):**
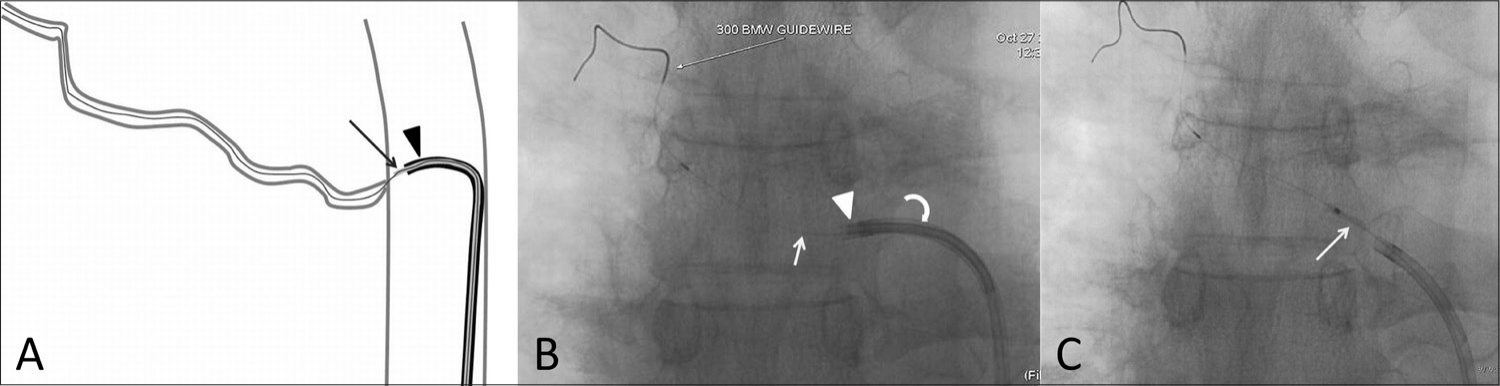
Technique of ostial dilatation. Line diagram (A) shows the technique of catheterizing and crossing of the stenotic ostium. An appropriately shaped diagnostic catheter (arrow) has been passed through a guiding catheter (arrowhead). A plain fluoroscopic image (B) shows a guide wire (arrow) being passed through the diagnostic catheter (arrowhead) which in turn has been passed through a guiding catheter (curved arrow). A subsequent image (C) shows a balloon (arrow) being used to dilate the ostium, after it has been exchanged for the diagnostic catheter

**Figure 3 (A,B):**
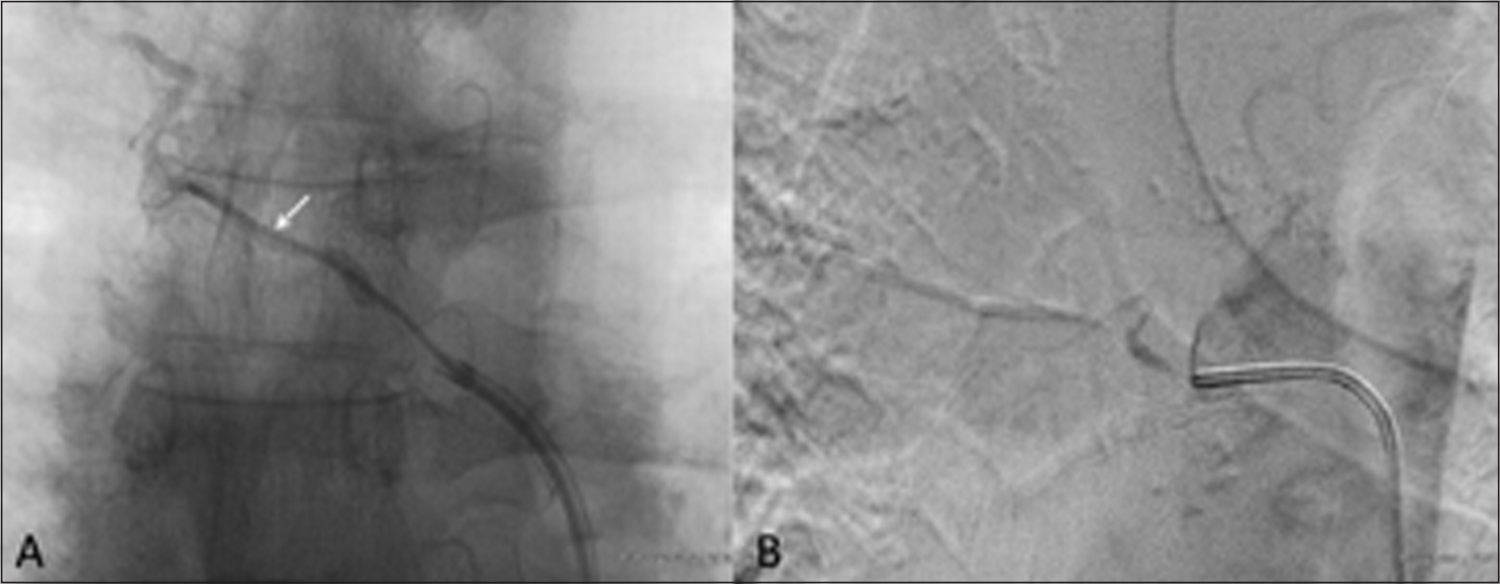
Embolization. DSA of the abnormal bronchial artery (A) shows a Progreat microcatheter (arrow) deep into the artery. Postprocedure angiogram (B) shows successful embolization of the vessel

A 61-year-old man with pulmonary tuberculosis presented with multiple episodes of recurrent hemoptysis, despite multiple sittings of multivessel embolization. The last sitting was 11 months ago. A multidetector CT scan showed showed three bronchial arteries, all showing stenosed ostia [Figure 1A]. Digital subtraction angiography (DSA) [Figure 1B] confirmed the findings. Two of these vessels with insignificant ostial stenosis were embolized. However, the patient rebled within 24 hours of the procedure. Check angiography showed no filling of the previously embolized vessels and it was decided to embolize the 3rd bronchial artery after dilating the ostial stenosis. A coaxial technique was used with a 6F double curve renal guiding catheter through which a 4F Cobra (Terumo Corporation, Tokyo, Japan) catheter was used to hook the diseased ostium of the culprit vessel [Figure 2A]. An exchange length 0.014” coronary wire was then navigated through the ostium into the bronchial artery [Figure 2B]. Following this, the Cobra catheter was removed and a 2.5 × 10 mm coronary balloon was tracked over the wire. The ostium was dilated [Figure 2C]. The renal guiding catheter was kept close to the ostium for test injection and stability of the wire. The balloon was then replaced by a Progreat microcatheter (Terumo Corporation, Tokyo, Japan) which was then passed easily into the bronchial artery. Injection through the microcatheter revealed no flow restriction [Figure 3A] and embolization was then performed with 500–710 μm polyvinylalcohol (PVA) particles (Cook Medical, Indiana, USA) [Figure 3B]. The patient has had no hemoptysis till date.

## Discussion

Remy and colleagues,[3] in 1973, first performed BAE, a procedure that has now established itself as a primary mode of therapy in patients with hemoptysis. BAE, however, is palliative and has a recurrence rate of 10–20%.[1] Surgery scores over BAE as it removes the source of bleeding and permits definitive treatment but is associated with a high mortality rate.[4–6] Surgery is usually reserved for those patients who continue to bleed despite repeated attempts at BAE. However, in patients declared unfit for anesthesia/surgery, repeated sittings of BAE may still be the only option available.

The inability to catheterize the bronchial arteries and instability of the catheter tip are frequent causes of technical failure.[1] This problem may be further compounded by ostial stenosis of the bronchial artery. Our literature search did not reveal any study discussing the incidence of bronchial artery stenosis or describing a technique such as ours for dilating a stenotic ostium prior to embolization. In our experience of 500 cases of BAE, we have seen stenotic ostia in about 5 patients, all in patients with atherosclerotic aortas. Predilatation of the stenotic ostium with a balloon introduced through the combination of a guiding catheter and a diagnostic catheter ensures forward flow and effective embolization of the distal vessel and prearteriolar bed. The only drawback of this innovative technique is that it is technically challenging and increases the procedure time as well as the cost.
